# Clinical utility of anti‐cytosolic 5’‐nucleotidase 1A antibody in idiopathic inflammatory myopathies

**DOI:** 10.1002/acn3.51294

**Published:** 2021-02-08

**Authors:** Chiseko Ikenaga, Andrew R. Findlay, Namita A. Goyal, Sarah Robinson, Jonathan Cauchi, Yessar Hussain, Leo H. Wang, Joshua C. Kershen, Brent A. Beson, Michael Wallendorf, Robert C. Bucelli, Tahseen Mozaffar, Alan Pestronk, Conrad C. Weihl

**Affiliations:** ^1^ Department of Neurology Washington University School of Medicine St. Louis Missouri USA; ^2^ Department of Neurology University of California Irvine California USA; ^3^ Austin Neuromuscular Center The University of Texas Dell Medical School Austin Texas USA; ^4^ Department of Neurology University of Washington Seattle Washington USA; ^5^ Integris Southwest Medical Center Oklahoma City Oklahoma USA; ^6^ Division of Biostatistics Washington University School of Medicine St. Louis Missouri USA

## Abstract

**Objective:**

To define the clinicopathologic features and diagnostic utility associated with anti‐cytosolic 5′‐nucleotidase 1A (NT5C1A) antibody seropositivity in idiopathic inflammatory myopathies (IIMs).

**Methods:**

Anti‐NT5C1A antibody status was clinically tested between 2014 and 2019 in the Washington University neuromuscular clinical laboratory. Using clinicopathologic information available for 593 patients, we classified them as inclusion body myositis (IBM), dermatomyositis, antisynthetase syndrome, immune‐mediated necrotizing myopathy (IMNM), nonspecific myositis, or noninflammatory muscle diseases.

**Results:**

Of 593 patients, anti‐NT5C1A antibody was found in 159/249 (64%) IBM, 11/53 (21%) dermatomyositis, 7/27 (26%) antisynthetase syndrome, 9/76 (12%) IMNM, 20/84 (24%) nonspecific myositis, and 6/104 (6%) noninflammatory muscle diseases patients. Among patients with IBM, anti‐NT5C1A antibody seropositive patients had more cytochrome oxidase‐negative fibers compared with anti‐NT5C1A antibody seronegative patients. Among 14 IBM patients initially negative for anti‐NT5C1A antibody, three patients (21%) converted to positive. Anti‐NT5C1A antibody seropositivity did not correlate with malignancy, interstitial lung disease, response to treatments in dermatomyositis, antisynthetase syndrome, and IMNM, or survival in IIMs.

**Interpretation:**

Anti‐NT5C1A antibody is associated with IBM. However, the seropositivity can also be seen in non‐IBM IIMs and it does not correlate with any prognostic factors or survival.

## Introduction

Inclusion body myositis (IBM) is an idiopathic inflammatory myopathy (IIM) that typically affects patients over the age of 50.^1^ Patients with IBM are clinically characterized by asymmetric finger flexion and knee extension weakness.^1^ In 2013, anti‐cytosolic 5′‐nucleotidase 1A (NT5C1A) antibody was detected in the sera of patients with IBM and recognized as a potential diagnostic marker for IBM.^2^, ^3^ Subsequently, the antibody was detected in patients with dermatomyositis, Sjögren's syndrome, and systemic lupus erythematosus.^4^, ^5^ This suggests that anti‐NT5C1A antibody can be detected in autoimmune diseases other than IBM, however, seropositivity for anti‐NT5C1A antibody in IIMs other than IBM has not been assessed within a large population. The relationship between seropositivity for anti‐NT5C1A antibody and other clinicopathologic features in IBM or dermatomyositis have been discussed and some report that seropositivity for anti‐NT5C1A antibody in IBM or juvenile myositis predict a more severe phenotype.^4^, ^10^, ^11^ This relationship has not been assessed in other IIMs.

Diagnostic testing for myositis specific and myositis associated antibodies is routinely performed in patients who are suspected of IIMs. Since 2014, the Washington University neuromuscular clinical laboratory has included anti‐NT5C1A antibody testing in its myositis panel or as an isolated antibody test. From over 100 distinct clinical institutions, 4987 patients, who were suspected of neuromuscular diseases, have had Clinical Laboratory Improvement Amendments‐certified clinical testing for anti‐NT5C1A antibody status through the Washington University neuromuscular clinical laboratory. In this study, we aimed to confirm the clinicopathologic correlation of anti‐NT5C1A antibody in IIMs using a cohort of clinically tested patients.

## Patients and methods

### Patients

We retrospectively identified 4987 patients, who underwent anti‐NT5C1A antibody testing at the Washington University in St. Louis (WashU) from 2014 to 2019, using the WashU myositis antibody database. In more than 90% of the patients other than IBM, this test was ordered as part of a “myositis panel.” Among them, the lists of patients from WashU, University of California, Irvine (UCI), The University of Texas Dell Medical School, University of Washington, and Integris Southwest Medical Center were made, respectively. The clinical chart, biopsy reports, and results of autoantibodies status of patients in the list were reviewed by the certified neuromuscular physicians in each center and those whose primary pathology is outside of skeletal muscle or whose final diagnosis was still under investigation were excluded. In this way, we recruited total 593 patients with primary muscle disease from WashU (*n* = 467), UCI (*n* = 75), The University of Texas Dell Medical School (*n* = 27), University of Washington (*n* = 14), and Integris Southwest Medical Center (*n* = 10).

The patients with IBM were classified as referring to the European Neuromuscular Center (ENMC) criteria.^13^ Twenty‐five IBM patients from a previous study were also included in our dataset.^11^ For the diagnosis of dermatomyositis, we also used the criteria defined in the ENMC international workshop.^14^ Antisynthetase syndrome was classified by the following, the presence of antiaminoacyl tRNA synthetase antibody and one or more of the following clinical features (Raynaud’s phenomenon, arthritis, interstitial lung disease, fever (not attributable to another cause), mechanic’s hands or biopsy finding of immune myopathy with perimysial pathology).^15^, ^16^

The patients with immune‐mediated necrotizing myopathy (IMNM) were seropositive for anti‐SRP antibody (*n* = 12) or anti‐HMGCR antibody (*n* = 53). We also included patients who were seronegative for anti‐SRP antibody and anti‐HMGCR antibody but with the muscle biopsy findings which were compatible with the diagnosis of IMNM (*n* = 11).^18^

Thus, the cohort included patients with IBM (*n* = 249), dermatomyositis (*n* = 53), antisynthetase syndrome (*n* = 27), and IMNM (*n* = 76). Patients that did not meet these criteria and had a muscle biopsy with endomysial or perimysial inflammation, major histocompatibility complex (MHC)‐class 1 up‐regulation on muscle fibers or complement C5b‐9 complex deposits on endomysial capillaries were categorized as nonspecific myositis (*n* = 84).

Patients with a noninflammatory muscle disease, who were genetically confirmed, were also included. The diagnoses of these 104 patients were valosin‐containing protein (VCP) ‐related multisystem proteinopathy (*n* = 26), limb‐girdle muscular dystrophy (*n* = 19), myotonic dystrophy (*n* = 9), mitochondrial myopathy (*n* = 5), facioscapulohumeral muscular dystrophy (*n* = 6), Becker muscular dystrophy (*n* = 5), McArdle's disease (*n* = 3), GNE myopathy (*n* = 3), myofibrillar myopathy (*n* = 3), Pompe disease (*n* = 2), dystroglycanopathy (*n* = 1), Poland syndrome (*n* = 1), and centronuclear myopathy (*n* = 1). Patients diagnosed with idiopathic rhabdomyolysis as evidenced by high serum CK, myoglobinuria, proximal weakness, and necrosis on muscle biopsy were also included (*n* = 20). In this way, a total of 593 patients with primary muscle diseases were recruited.

### Serological testing for autoantibodies

Anti‐NT5C1A antibody testing was clinically performed by the Washington University Neuromuscular Laboratory via Western blotting as previously described.^11^ The myositis panel in Washington University Neuromuscular Laboratory also includes anti‐Jo‐1 antibody, anti‐HMGCR antibody, anti‐SRP antibody, and anti‐MDA5 antibody.^19^ Serums were tested and interpreted blindly without knowledge of the diagnosis. As for the other myositis specific antibodies, they were also evaluated through panel testing by clinical laboratories in Oklahoma or California. These data were extracted from the clinical chart.

### Myopathologic analysis

The myopathological findings were retrieved from previous pathology reports. The muscle biopsies were taken for diagnostic purposes and read by the neuromuscular specialists in each institute. Muscle tissue sections were subjected to routine histochemical and immunohistochemical analyses including MHC‐class 1, CD8, CD4, CD20, C5b‐9 complement (membrane attack complex), SMI‐31, and LC3 staining. We defined endomysial inflammation as endomysial inflammatory infiltrate surrounding or invading non‐necrotic muscle fibers. The percentages of cytochrome oxidase (COX)‐negative and succinate dehydrogenase‐positive muscle fibers were determined by photographing a random field with a 10x objective and counting 200 fibers by C.I. and double‐checked by C.C.W. A cutoff value of 1.0% was used for qualitative analysis of COX‐negative fibers as with our previous report.^20^

### Definition of clinical features and assessment of responses to immunotherapy

Dermatologic features were defined by clinical description (i.e., Heliotrope rash, Gottron’s sign, V‐sign, shawl sign, calcinosis cutis, and mechanic’s hands), skin biopsy data, and/or expert opinion of a dermatologist. Inflammatory arthritis was defined by imaging features and/or expert opinion of a rheumatologist. Interstitial lung disease was defined by imaging features, pulmonary function tests, biopsy data, and/or expert opinion of a pulmonologist. Response to treatment was determined for patients followed by more than 1 year as: effective, muscle weakness had diminished and the level of activities of daily living had improved to the same as premorbid level; no worsening, some symptoms had improved but there were still several symptoms or abnormality on blood test; worsening, muscle weakness was progressive and there was no improvement with any immunosuppressive treatments.

### Statistical analyses

Fisher exact test compared categorical variables and t‐test compared continuous variables. We considered two‐sided *p* values less than 0.05 as statistically significant. To control for multiple comparisons, we applied Bonferroni correction to provide an adjusted threshold for significance in Table 3 and Table S2. Data were analyzed using R, version 3.6.1 (The R Foundation). The influence of anti‐NT5C1A antibody status on survival was assessed using Kaplan–Meier curves and log‐rank testing using GraphPad Prism, version 8.4.3 (GraphPad Software). Date of disease onset was set as a start of the surveillance and the date of death or the latest date at the outpatient clinic were set as an endpoint.

## Results

Among the 593 patients with primary muscle disease, 212 patients (36%) were seropositive for the anti‐NT5C1A antibody. Among the 489 patients with IIMs, there were 206 patients (42%), who were seropositive for anti‐NT5C1A antibody. We compared anti‐NT5C1A antibody seropositive patients and seronegative patients among these 489 patients (Table 1). There was no significant difference as to the ratio of male to female, age at onset, or presence of perimysial inflammation. In contrast, anti‐NT5C1A antibody seropositive patients had a lower serum CK (*P* < 0.01), higher frequency of antinuclear antibody seropositivity (59% vs. 18%; *P* < 0.01), higher frequency of dysphagia (45% vs. 28%; *P* < 0.01), and the presence of MHC‐class1 expression (87% vs. 70%; *P* < 0.01), compared with seronegative patients. To see if these differences were due to the overrepresentation of IBM patients in the anti‐NT5C1A antibody seropositive group, we performed a similar analysis that removed the 249 patients with IBM and thus looked at 240 non‐IBM IIMs (Table 2). Similar to the previous group, anti‐NT5C1A antibody seropositive non‐IBM IIM patients had a lower serum CK (*P* = 0.02) and a higher frequency of antinuclear antibody (45% vs. 18%; *P* < 0.01). Notably, when IBM patients were removed from the analysis, there was no significant difference in dysphagia or MHC‐class1 expression.

**TABLE 1:** Clinicopathologic features of patients with idiopathic inflammatory myopathies

	Patients with idiopathic inflammatory myopathies (*n* = 489)		
	Anti‐NT5C1A positive	Anti‐NT5C1A negative	*P*
Number of patients	206 (42)	283 (58)	
Sex (M:F)	105:101	139:144	0.71
Age at onset, mean (SD), years	57.8 (10.1)	55.5 (14.8)	0.05
Maximum CK level, median, IU/L	451	479	**<0.01**
Dysphagia, no. (%)	89/199 (45)	71/255 (28)	**<0.01**
Antinuclear antibody, no. (%)	43/73 (59)	28/152 (18)	**<0.01**
Other antibodies^1^, no. (%)	49/138 (36)	113/250 (45)	0.07
Muscle biopsy findings, no. (%)			
Perimysial inflammation	59/152 (39)	84/233 (36)	0.59
MHC‐class1 upregulation	102/117 (87)	139/199 (70)	**<0.01**

CK, creatine kinase; MHC, major histocompatibility complex.

^1^ Antibodies against MDA5, TIF1γ, NXP2, Mi2, Jo‐1, EJ, OJ, HMGCR, SRP, SS‐A, SS‐B, PM‐Scl, and U1snRNP were included. The *P*‐values less than 0.05 are marked in bold.

**TABLE 2:** Clinicopathologic features of patients with idiopathic inflammatory myopathies other than IBM

	Patients with idiopathic inflammatory myopathies other than IBM (*n* = 240)		
	Anti‐NT5C1A positive	Anti‐NT5C1A negative	*P*
Number of patients	47 (20)	193 (80)	
Sex (M:F)	13:34	85:108	0.05
Age at onset, mean (SD), years	54.3 (14.0)	53.3 (16.4)	0.67
Maximum CK level, median, IU/L	848	952	**0.02**
Dysphagia, no. (%)	7/41 (17)	31/170 (18)	>0.99
Antinuclear antibody, no. (%)	14/31 (45)	22/121 (18)	**<0.01**
Other antibodies^1^, no. (%)	22/47 (47)	96/193 (50)	0.75
Muscle biopsy findings, no. (%)			
Perimysial inflammation	15/38 (39)	66/158 (42)	0.86
MHC‐class1 upregulation	24/32 (75)	88/138 (64)	0.30

CK, creatine kinase; MHC, major histocompatibility complex.

^1^ Antibodies against MDA5, TIF1γ, NXP2, Mi2, Jo‐1, EJ, OJ, HMGCR, SRP, SS‐A, SS‐B, PM‐Scl, and U1snRNP were included. The *P*‐values less than 0.05 are marked in bold.

As expected, independent of anti‐NT5C1A antibody status, patients with IBM were significantly older (59.4 ± 8.7 vs. 53.5 ± 15.9 years old; *P* < 0.01) and male 59% as compared to 41% in the non‐IBM cohort (*P* < 0.01, Table S1). In addition, IBM patients showed lower serum CK (*P < *0.01), dysphagia (50% vs. 18%; *P < *0.01), and higher frequency of MHC‐class1 expression (88% vs. 66%; *P < *0.01).

Among the 104 noninflammatory muscle disease, 26 patients with VCP‐related multisystem proteinopathy showed higher rate of anti‐NT5C1A antibody seropositivity (*n* = 4, 15%) compared with that in 78 other noninflammatory muscle diseases (*n* = 2, 3%; *P* = 0.03). The percentage of anti‐NT5C1A antibody seropositive patients with IIMs is significantly higher than that of patients with noninflammatory muscle disease (42% vs. 6%; *P < *0.01).

### Clinicopathologic correlation of anti‐NT5C1A antibody in patients with dermatomyositis, antisynthetase syndrome, and IMNM

We compared clinicopathologic features and responsiveness to immunosuppressive treatments in non‐IBM IIM patients who underwent anti‐NT5C1A antibody testing. Among patients with dermatomyositis (*n* = 53), antisynthetase syndrome (*n* = 27), and IMNM (*n* = 76), 11 (21%), 7 (26%), and 9 (12%) patients were seropositive for anti‐NT5C1A antibody, respectively (Table S2). Anti‐NT5C1A antibody seropositive dermatomyositis patients had a higher frequency of calcinosis cutis (55% vs. 8%; *P* < 0.01). Among the nine patients who had calcinosis cutis, seven patients (anti‐NT5C1A antibody seropositive (*n* = 4) and anti‐NT5C1A antibody seronegative (*n* = 3)) were tested for anti‐NXP2 antibody and anti‐SAE antibody through myositis panel test. One anti‐NT5C1A antibody‐positive patient was seropositive for anti‐TIF1γ antibody, whereas two anti‐NT5C1A antibody‐negative patients were seropositive for anti‐NXP2 antibody or anti‐Mi2 antibody.

There was no biopsy finding which seems to have correlate with the seropositivity of anti‐NT5C1A antibody in dermatomyositis, antisynthetase syndrome, and IMNM. Notably, in dermatomyositis or antisynthetase syndrome, there was no significant difference between anti‐NT5C1A antibody seropositive and seronegative patients as for the association with interstitial lung disease. Interestingly, among patients with dermatomyositis, antisynthetase syndrome, and IMNM that had an associated malignancy, all were seronegative for anti‐NT5C1A antibody. Also, anti‐NT5C1A antibody status did not correlate with treatment response among these patients.

### Clinicopathologic correlation of anti‐NT5C1A antibody in patients with IBM and follow‐up of their anti‐NT5C1A antibody status

Among 249 patients with IBM, 64% were seropositive for anti‐NT5C1A antibody. In other words, the sensitivity of the antibody for the diagnosis of IBM was 64%. Anti‐NT5C1A antibody seropositivity was 85% specific for IBM among all patients with muscle diseases, and 80% specific for IBM among IIMs. Anti‐NT5C1A antibody seropositive IBM patients had a higher frequency of finger flexion weakness (91% vs. 76%; *P* < 0.01), but no significant differences were seen with regard to age at onset, maximum CK level, dysphagia, or complications of other autoimmune diseases/sarcoidosis/malignancy (Table 3, Table S3). Anti‐NT5C1A antibody seropositive IBM patients also had significantly more COX‐negative fibers (3.2 ± 3.9 vs. 2.1 ± 2.3; *P* = 0.04). There were no other significant differences in muscle biopsy features. There were 36 patients with IBM whose anti‐NT5C1A antibody status had been retested at an interval of greater than 1 year. Anti‐NT5C1A antibody status did not change in 32 patients (89%). Among the 14 patients who were negative for anti‐NT5C1A antibody, three patients (21%) converted to positive. There was no significant difference between these three patients and those whose antibody status remained negative regarding age at onset (49.3 ± 11.4 vs. 56.4 ± 10.8; *P* = 0.41). Among the 22 patients who were seropositive for anti‐NT5C1A antibody, there was a 61‐year‐old male patient whose anti‐NT5C1A antibody status changed to negative despite the progression of grip difficulty and proximal leg weakness.

**TABLE 3:** Clinicopathologic features of 249 patients with IBM according to anti‐NT5C1A antibody status

	Anti‐NT5C1A positive	Anti‐NT5C1A negative	*P* ^1^
Number of patients	159 (64)	90 (36)	
Sex (M:F)	92:67	54:36	0.80
Age at onset, mean (SD), years	58.8 (8.3)	60.3 (9.3)	0.21
Onset to biopsy, mean (SD), years	5.2 (4.6)	5.6 (5.1)	0.60
Maximum CK level, median, IU/L	425	308	0.11
Dysphagia	82/158 (52)	40/85 (47)	0.50
Knee extension weakness (≥ hip flexion weakness)	121/159 (76)	69/90 (77)	>0.99
Finger flexion weakness (> shoulder abduction weakness)	145/159 (91)	68/90 (76)	**<0.01**
Muscle biopsy findings			
Rimmed vacuoles	93/159 (58)	63/90 (70)	0.08
Protein accumulation	71/95 (75)	53/64 (83)	0.25
Endomysial inflammatory infiltrate	147/155 (95)	79/89 (89)	0.12
Focal invasion of non‐necrotic fibers	84/117 (72)	43/71 (61)	0.15
MHC‐class1 upregulation	78/85 (92)	51/61 (84)	0.19
Qualitative analysis of COX‐negative fibers^2^	86/122 (70)	52/76 (68)	0.75
COX‐negative fibers, mean (SD), %^2^	3.2 (3.9)	2.1 (2.3)	**0.04**
SDH‐positive fibers, mean (SD), %^2^	2.2 (2.9)	2.0 (2.8)	0.69

CK, creatine kinase; COX, cytochrome oxidase; IBM, inclusion body myositis; SDH, succinate dehydrogenase. Values are no. (%) unless otherwise indicated.

^1^ Bonferroni corrected significance thresholds for clinical features and biopsy findings were 0.003 and 0.007, respectively. The *P*‐values less than 0.05 are marked in bold.

^2^ The percentages of COX‐negative and SDH‐positive muscle fibers were determined by photographing a random field with a 10x objective and counting 200 fibers. We set cutoff value as 1.0% for qualitative analysis of COX‐negative fibers.

### Estimation of survival

**FIGURE 1:**
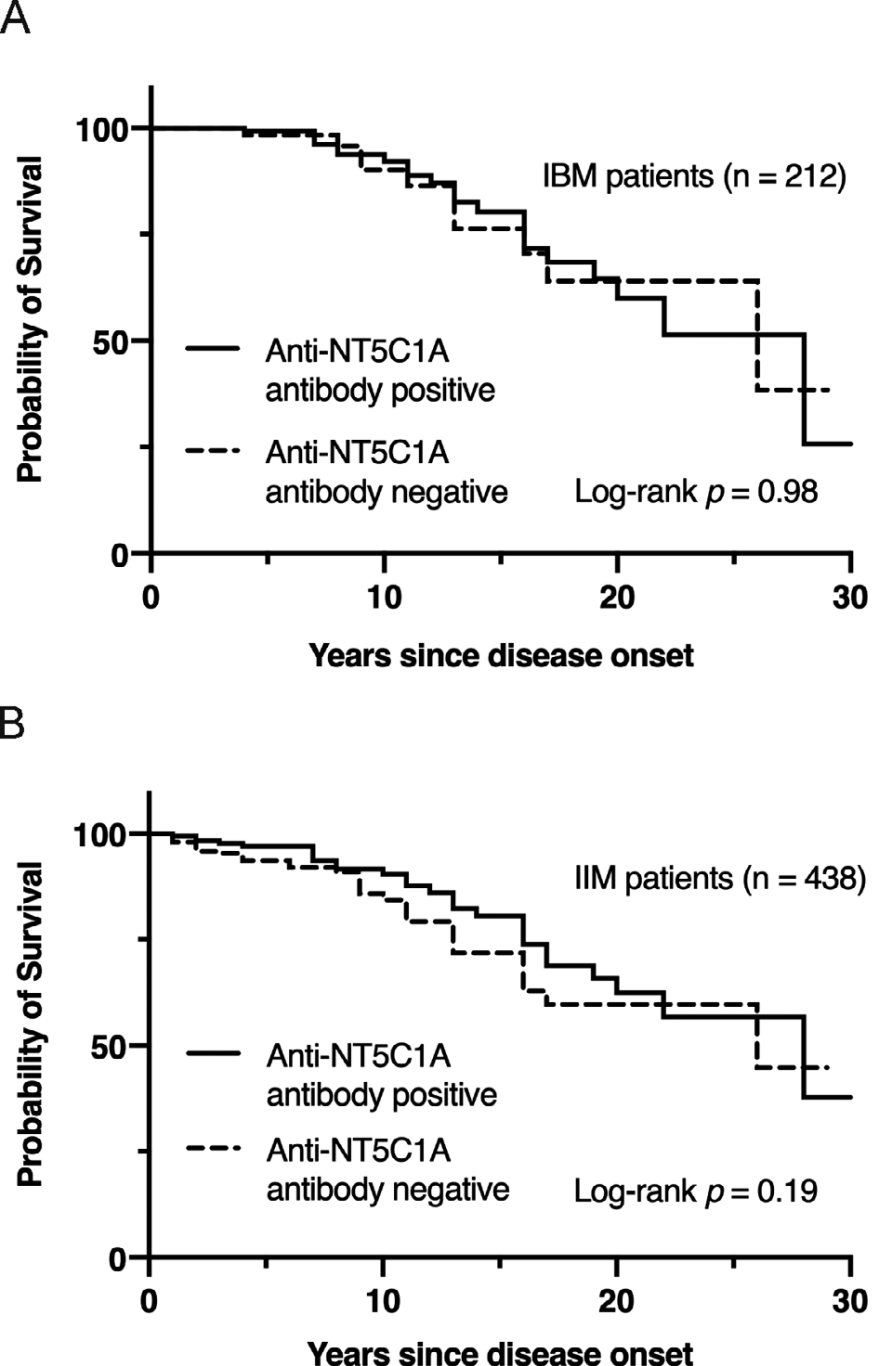
Kaplan–Meier survival curves depending on the anti‐NT5C1A antibody status. (A) Kaplan–Meier survival curves of 212 patients with inclusion body myositis. (B) Kaplan–Meier survival curves of 438 patients with idiopathic inflammatory myopathies.

Among the 489 IIM patients, both the date of disease onset and the date of last follow‐up/date of death were available on 438 patients, which included 212 patients with IBM. In total, they were followed for 3472 patient‐years and 61 patients expired. There was no significant difference between the anti‐NT5C1A antibody‐positive and anti‐NT5C1A antibody‐negative patients’ Kaplan–Meier curves (Fig. 1; log‐rank *P* = 0.98 in patients with IBM and log‐rank *P* = 0.19 in patients with IIMs). Subcohort analysis for two individual sites representing the two largest cohorts (WashU and UCI) demonstrated variability in the survival rate of anti‐NT5C1A antibody‐positive patients with UCI being lower than all patients. (Figure S1, A: *P* = 0.73 in patients in WashU, B: *P* = 0.21 in patients in UCI).

## DISCUSSION

In this study, we describe the clinicopathologic correlation of anti‐NT5C1A antibody in patients with IIMs. Notably, anti‐NT5C1A antibody positivity was seen in IBM, antisynthetase syndrome, IMNM, and dermatomyositis. Dermatomyositis patients who were seropositive for anti‐NT5C1A antibody showed a higher frequency of calcinosis cutis irrespective of the presence of anti‐NXP2 antibody or anti‐SAE antibody. Calcinosis cutis has been considered to be caused by chronic inflammation and vascular hypoxia, however, the detailed pathophysiology remains poorly understood.^21^ However, one previous study of 159 dermatomyositis patients found no significant difference including calcinosis between seropositive and seronegative patients.^7^ The different results may be derived from the difference in method to detect the antibody, inclusion criteria, or the number of patients evaluated. This inconsistency suggests that there is no correlation between the seropositivity for anti‐NT5C1A antibody and the clinical features, which were checked in this study.

Although there were patients who were seropositive for anti‐NT5C1A antibody among those with antisynthetase syndrome or IMNM, their clinicopathologic features were not different from those in seronegative patients. Anti‐NT5C1A antibody positivitity was not associated with malignancy and interstitial lung disease, which are features important for patient management and prognosis in patients with dermatomyositis, antisynthetase syndrome, and IMNM. Thus, in these myositis, anti‐NT5C1A antibody is not an indicator of disease severity.

Previously, seropositivity for anti‐NT5C1A antibody has been reported to correlate with dysphagia and a more severe phenotype in IBM with a higher adjusted mortality risk.^11^, ^12^ However, in this study, there was no consistent correlation between anti‐NT5C1A antibody seropositivity and Kaplan–Meier survival curves, although anti‐NT5C1A antibody‐positive patients tended to have higher mortality risk in some smaller cohorts, which is consistent with a previous report that seropositive patients have a more severe phenotype.^11^ Regarding to dysphagia, anti‐NT5C1A antibody seropositive IIMs patients had a higher frequency of dysphagia. However, dysphagia was not significantly different between anti‐NT5C1A antibody seropositive patients and seronegative patients in IBM. Another report also failed to find a difference between anti‐NT5C1A antibody‐positive and ‐negative patients.^22^ These previous studies did not show the relationship between seropositivity and distal upper limb/grip weakness, in our study, seropositive IBM patients showed more frequent involvement of finger flexion weakness. Again, the difference may be derived from the difference in method to detect the antibody, inclusion criteria, or the number of patients evaluated.

In IBM patients, anti‐NT5C1A antibody seropositivity has been previously reported to be associated with an increase in biopsies containing COX‐negative fibers similar to our findings.^12^ In contrast to a previous report that found anti‐NT5C1A antibody seropositive patients had a lower frequency of rimmed vacuoles in IBM, there was no difference as to the presence of the rimmed vacuoles between anti‐NT5C1A antibody‐positive and ‐negative patients in this study.^7^ These findings suggest that anti‐NT5C1A antibody status may correlate more strongly with mitochondrial dysfunction, rather than degenerative pathways represented by the presence of rimmed vacuoles. Interestingly, the seropositivity for anti‐NT5C1A antibody in VCP‐related multisystem proteinopathy was higher than that in other noninflammatory muscle diseases. Some patients with clinico‐pathologically defined IBM have been found to have pathogenic VCP mutations.^23^, ^24^ There may be a common pathway shared with IBM patients and patients with VCP‐related multisystem proteinopathy, that involves stimulating the production of anti‐NT5C1A antibodies.

Among patients with muscle diseases, the seropositivity of anti‐NT5C1A antibody was 64% sensitive for the diagnosis of IBM. This result compares with the results of previous studies, which suggested the range of sensitivity was 33% to 80%.^2^, ^3^, ^5^, ^6^, ^7^ The sensitivity of anti‐NT5C1A antibody for IBM with western blot was 61% in a previous study and 64% in this study.^7^ Among studies using more than 10 samples from patients with IBM, the sensitivities of western blotting were consistently higher than that of ELISA assay unless a combination of ELISA assays was used to detect IgM, IgA, and IgG anti‐NT5C1A antibodies.^25^ However, this high sensitivity was also applied to those in other IIMs, which decreased the specificity for the diagnosis of IBM in this study. In adult patients with dermatomyositis, the seropositivity of anti‐NT5C1A antibody was reported to be 0% to 15 %, whereas in this study it was 21%.^2^, ^3^, ^6^, ^7^, ^8^, ^10^

In clinical practice, myositis antibody panels are often ordered in patients with a suspected IIM. Indeed, more than 90% of the patients other than IBM were tested for anti‐NT5C1A antibody through panel testing. Yet it is important to note that in IBM patients whose anti‐NT5C1A antibody was initially negative at the beginning, 21% converted to positive. Retesting anti‐NT5C1A antibody status may be clinically helpful when a patient is suspected of a treatable IIM but does not show a response to immunomodulation.^26^, ^27^

This study has limitations. First, there may be a selection bias for patients with IIMs other than IBM. Specifically, some patients were reflexively tested for the anti‐NT5C1A antibody when the physician ordered a myositis panel even though they clinically did not meet the criteria for IBM. Others were intentionally tested for anti‐NT5C1A antibody after a diagnosis of IBM was determined via both clinical and pathologic studies. Second, this is a retrospective study of clinical records from five neuromuscular centers and some features, which were not described in the clinical charts, may not have been adequately captured. We used a common format to fill in the clinicopathological information in order to minimize the gap between the institutes regarding the way information was collected.

Anti‐NT5C1A antibody testing is a worthwhile addition to a myositis panel for initial assessments to eliminate noninflammatory muscle diseases from diagnoses. However, the seropositivity can be widely seen in IIMs and it does not correlate with any prognostic factors or survival. Thus, clinicians should cautiously interpretate anti‐NT5C1A antibody status and correlate the result with the clinical exam and biopsy findings.

## Authors’ Contributions

CI, ARF, NAG, RCB, TM, AP, and CCW made contributions to conception and design of the study. CI, ARF, NAG, SR, JC, YH, LHW, JCK, BAB, TM, AP, and CCW carried out acquisition of data. CI, SR, MW, and CCW were involved in analysis of data. CI, ARF, and CCW were involved in draft manuscript preparation. All authors were involved in revising the draft critically for important intellectual content and approved the final manuscript to be submitted.

## Conflict of Interest

CI, ARF, SR, JC, YH, JCK, BAB, MW, and RCB report nothing to declare. NAG has received research support from Brainstorm Cell Therapeutics, Cytokinetics, Fulcrum, Kezar, Novartis, Octapharma, Orion, and Orphazyme. She has served on Advisory Boards for Acceleron, Alexion, Argenx, Biogen, CSL Behring, Cytokinetics, MT Pharma, Novartis, Sanofi Genzyme, Sarepta. In relation to these activities, she has received travel reimbursement and honoraria. She has also served on the speaker’s bureau for CSL. LHW reports grants from Muscular Dystrophy Association, grants from Friends of FSH Research.TM has served on advisory boards for Abbvie, Alexion, Amicus, Argenx, Audentes, Sanofi‐Genzyme, Sarepta, and Spark Therapeutics. In relation to these activities, he has received travel reimbursement and honoraria. He has also served on the speaker’s bureau for Alexion, CSL, Grifols, and Sanofi‐Genzyme. He serves on the medical advisory board for the Myositis Association, Neuromuscular Disease Foundation, Myasthenia Gravis Foundation of California, and Myasthenia Gravis Foundation of America. He has received travel funding from the Myositis Association and the Neuromuscular Disease Foundation. He has received research funding from the Myositis Association, the Muscular Dystrophy Association, and from the following sponsors: Alexion, Amicus, Argenx, Audentes, Bristol‐Myers‐Squib, Cartesian Therapeutics, Grifols, Momenta, Ra Pharmaceuticals, Sanofi‐Genzyme, Spark Therapeutics, UCB, and Valerion. He serves on the data safety monitoring board for Acceleron. AP has a patent TS‐HDS and other antibody testing methods with royalties paid. He received research support from Acceleron, Idera, Knopp, Cytokinetics, Biogen Idec, Fulcrum, Genzyme, Ionis, Sanofi, Ultragenyx, and personal fees from Athena. He holds stock in Johnson & Johnson. CCW reports grants and personal fees from Sarepta, personal fees from Acceleron, Casma therapeutics, and ML Bio.

## Supporting information


**Figure S1**. Kaplan–Meier survival curves depending on the anti‐NT5C1A antibody status in patients with inclusion body myositis. A: Kaplan–Meier survival curves of 154 patients in the Washington University in St. Louis. B: Kaplan–Meier survival curves of 58 patients in the University of California, Irvine.
**Table S1**. Clinicopathologic features of patients with IBM compared with non‐IBM patients with idiopathic inflammatory myopathies
**Table S2**. Clinicopathologic features of patients with dermatomyositis, antisynthetase syndrome, and immune‐mediated necrotizing myopathy according to anti‐NT5C1A antibody status
**Table S3**. Diagnoses with ENMC criteria and complications of patients with IBM according to anti‐NT5C1A antibody status

## Data Availability

The deidentified patient data that support the findings of this study are available from the corresponding author, upon reasonable request. All studies were approved by the Human Studies Committee at Washington University in St. Louis (WUIRB#201101855). All patients were reviewed in a de‐identified manner corresponding to their clinically obtained serum number.
