# Supplementary material for: Clinical utility of anti‐cytosolic 5’‐nucleotidase 1A antibody in idiopathic inflammatory myopathies
Source: Ann Clin Transl Neurol. 2021 Feb 8;8(3):571–8. doi: 10.1002/acn3.51294 (PMC7951108; doi:10.1002/acn3.51294)

| **Supplementary Table 1. Clinicopathologic features of patients with IBM compared with non-IBM patients with idiopathic inflammatory myopathies** | | | |
| --- | --- | --- | --- |
|  | Patients with idiopathic inflammatory myopathies  (n = 489) | | |
|  | IBM | non-IBM | *p*^a^ |
| Number of patients | 249 (51) | 240 (49) |  |
| Sex (M:F) | 146:103 | 98:142 | **< 0.01** |
| Age at onset, mean (SD), years | 59.4 (8.7) | 53.5 (15.9) | **< 0.01** |
| Maximum CK level, median, IU/L | 372 | 922 | **< 0.01** |
| Dysphagia, no. (%) | 122/243 (50) | 38/211 (18) | **< 0.01** |
| Antinuclear antibody, no. (%) | 35/73 (48) | 36/152 (24) | **< 0.01** |
| Other antibodies^b^, no. (%) | 44/148 | 118/240 (49) | **< 0.01** |
| Muscle biopsy findings, no. (%) |  |  |  |
| Perimysial inflammation | 62/189 (33) | 81/196 (41) | 0.09 |
| MHC-class1 upregulation | 129/146 (88) | 112/170 (66) | **< 0.01** |
| Qualitative analysis of COX negative fibers^c^ | 138/198 (70) | 29/165 (18) | **< 0.01** |
| COX negative fibers, mean (SD), % ^c^ | 2.8 (3.5) | 0.2 (0.5) | **< 0.01** |
| SDH positive fibers, mean (SD), % ^c^ | 2.2 (2.8) | 0.3 (1.0) | **< 0.01** |
| Abbreviations: IBM = inclusion body myositis; CK = creatine kinase; MHC = major histocompatibility complex; COX = cytochrome oxidase; SDH = succinate dehydrogenase.  ^a^ The *p*-values less than 0.05 are marked in bold. ^b^Antibodies against MDA5, TIF1γ, NXP2, Mi2, Jo-1, EJ, OJ, HMGCR, SRP, SS-A, SS-B, PM-Scl, and U1snRNP were included. ^c^ The percentages of COX negative and SDH positive muscle fibers were determined by photographing a random field with a 10x objective and counting 200 fibers. We set cutoff value as 1.0% for qualitative analysis of COX negative fibers. | | | |

| **Supplementary Table 2. Clinicopathologic features of patients with dermatomyositis, antisynthetase syndrome, and immune mediated necrotizing myopathy according to anti-NT5C1A antibody status** | | | | | | | | | |
| --- | --- | --- | --- | --- | --- | --- | --- | --- | --- |
|  | Dermatomyositis  (n = 53) | | | Antisynthetase syndrome  (n = 27) | | | Immune mediated necrotizing myopathy (n = 76) | | |
|  | Anti-NT5C1A positive | Anti-NT5C1A negative | *p*^a^ | Anti-NT5C1A positive | Anti-NT5C1A negative | *p*^a^ | Anti-NT5C1A positive | Anti-NT5C1A negative | *p*^a^ |
| Number of patients | 11 (21) | 42 (79) |  | 7 (26) | 20 (74) |  | 9 (12) | 67 (88) |  |
| Sex (M:F) | 1:10 | 12:30 | 0.26 | 3:4 | 8:12 | > 0.99 | 2:7 | 32:35 | 0.18 |
| Age at onset, mean (SD), years | 50.2 (13.8) | 51.3 (15.2) | 0.82 | 56.6 (11.3) | 48.9 (13.8) | 0.17 | 57.6 (17.8) | 57.9 (16.0) | 0.96 |
| Maximum CK level, median, IU/L | 737 | 212 | 0.58 | 2858 | 1153 | 0.24 | 2565 | 3576 | 0.29 |
| Dysphagia | 3/10 (30) | 17/41 (41) | 0.72 | 4/7 (57) | 7/19 (37) | 0.41 | 2/9 (22) | 12/64 (19) | > 0.99 |
| Fever | 0/10 | 2/38 (5) | > 0.99 | 0/7 | 3/19 (16) | 0.54 | NA | NA | NA |
| Dyspnea | 0/10 | 7/37 (19) | 0.32 | 2/7 (29) | 10/17 (59) | 0.37 | 3/9 (33) | 10/64 (16) | 0.19 |
| Heliotrope rash | 3/10 (30) | 10/42 (24) | 0.70 | 0/7 | 0/19 | > 0.99 | NA | NA | NA |
| Gottron's sign | 5/10 (50) | 16/42 (38) | 0.50 | 0/7 | 0/19 | > 0.99 | NA | NA | NA |
| Calcinosis cutis | 6/11 (55) | 3/40 (8) | **< 0.01** | NA | NA | NA | NA | NA | NA |
| Mechanic's hands | 1/10 (10) | 3/38 (8) | > 0.99 | 0/7 | 6/19 (32) | 0.15 | NA | NA | NA |
| Other skin symptom | 8/11 (73) | 36/42 (86) | 0.37 | 0/7 | 6/19 (32) | 0.15 | NA | NA | NA |
| Raynaud's phenomenon | 2/10 (20) | 2/41 (5) | 0.17 | 0/7 | 2/19 (11) | > 0.99 | NA | NA | NA |
| Arthritis | 2/10 (20) | 11/41 (27) | > 0.99 | 4/7 (57) | 7/19 (37) | 0.41 | NA | NA | NA |
| Weakness | 10/11 (91) | 35/42 (83) | > 0.99 | 6/7 (86) | 14/17 (82) | > 0.99 | 7/8 (88) | 56/60 (93) | 0.48 |
| Interstitial lung disease | 0/10 | 9/41 (22) | 0.18 | 2/7 (29) | 11/19 (58) | 0.38 | NA | NA | NA |
| Malignancy^b^ | 0/11 | 5/41 (12)^c^ | 0.57 | 0/7 | 1/19 (5)^d^ | > 0.99 | 0/9 | 7/63 (11)^e^ | 0.58 |
| Complications of other autoimmune disease |  |  |  |  |  |  |  |  |  |
| Sjögren syndrome | 0 | 1/42 (2) | > 0.99 | 0 | 0 | > 0.99 | 0 | 1/67 (1) | > 0.99 |
| Rheumatoid arthritis | 0 | 1/42 (2) | > 0.99 | 0 | 3/20 (15) | 0.55 | 0 | 1/67 (1) | > 0.99 |
| Systemic lupus erythematosus | 1/11(9) | 1/42 (2) | 0.38 | 0 | 1/20 (5) | > 0.99 | 1/9 (13) | 2/67 (3) | 0.35 |
| Scleroderma | 2/11(18) | 1/42 (2) | 0.11 | 0 | 1/20 (5) | > 0.99 | 0 | 0 | > 0.99 |
| Hypothyroidism | 3/11(27) | 6/42 (14) | 0.37 | 0 | 3/20 (15) | 0.55 | 3/9 (33) | 5/67 (7) | 0.05 |
| Muscle biopsy findings |  |  |  |  |  |  |  |  |  |
| MHC-class1 upregulation | 6/7 (86) | 20/24 (83) | > 0.99 | 3/4 (75) | 11/12 (92) | 0.45 | 4/7 (57) | 27/47 (57) | > 0.99 |
| Perimysial inflammatory infiltrate | 4/8 (50) | 13/27 (48) | > 0.99 | 3/6 (50) | 9/15 (60) | > 0.99 | 2/8 (25) | 13/53 (25) | > 0.99 |
| Immune myopathies with perimysial pathology | 3/8 (38) | 5/27 (19) | 0.35 | 3/6 (50) | 7/15 (47) | > 0.99 | 2/8 (25) | 6/53 (11) | 0.28 |
| Sarcolemmal deposits of C5b9 | 0/4 | 9/19 (47) | 0.13 | 2/4 (50) | 4/9 (44) | > 0.99 | 3/7 (43) | 20/43 (47) | > 0.99 |
| Normalization of CK level | 8/10 (80) | 22/31 (71) | 0.70 | 5/6 (83) | 11/14 (79) | > 0.99 | 4/9 (44) | 32/55 (58) | 0.49 |
| Responses to immunosuppressive treatments^f^ |  |  |  |  |  |  |  |  |  |
| Effective | 8/11 (73) | 23/37 (62) | 0.72 | 3/7 (43) | 6/14 (43) | > 0.99 | 3/9 (33) | 22/53 (42) | 0.73 |
| No worsening | 3/11 (27) | 8/37 (22) | NA | 4/7 (57) | 6/14 (43) | NA | 3/9 (33) | 18/53 (34) | NA |
| Worsening | 0/11 | 6/37 (16) | NA | 0/7 | 2/14 (14) | NA | 3/9 (33) | 13/53 (25) | NA |
| Abbreviations: CK = creatine kinase; NA= Not applicable; MHC = major histocompatibility complex. Values are no. (%) unless otherwise indicated. ^a^ Bonferroni corrected significance thresholds for clinical features and biopsy findings were 0.002 and 0.013, respectively. The *p*-value less than 0.05 is marked in bold. ^b^Not all the patients were tested for CT, PET, or tumor markers as cancer screening. ^c^ Colon cancer (n = 2), bladder cancer, basal cell carcinoma of the neck, and adenocarcinoma of unknown primary. ^d^ Spinal cord tumor. ^e^ Prostate cancer (n = 2), breast cancer, Hodgkin's lymphoma, non-Hodgkin's lymphoma, tonsil squamous cell carcinoma, and nasopharyngeal basaloid squamous cell carcinoma. ^f^ Patients who showed “effective” response were compared with those who showed “no worsening” or “worsening” responses. | | | | | | | | | |

| **Supplementary Table 3. Diagnoses with ENMC criteria and complications of patients with IBM according to anti-NT5C1A antibody status** | | | |
| --- | --- | --- | --- |
|  | Anti-NT5C1A positive  (n = 159) | Anti-NT5C1A negative  (n = 90) | *p*^a^ |
| Diagnosis with ENMC criteria (Rose *et al.* 2013) |  |  |  |
| Clinico-pathologically defined IBM | 74/159 (47) | 51/90 (57) | 0.15 |
| Clinically defined IBM | 56/159 (35) | 22/90 (24) | 0.09 |
| Probable IBM | 29/159 (18) | 17/90 (19) | > 0.99 |
| Complications of autoimmune diseases other than IBM | 23/157 (15)^b^ | 18/82 (22) | 0.21 |
| Sjögren syndrome | 7/157 (4) | 4/82 (5) | > 0.99 |
| Rheumatoid arthritis | 2/157 (1) | 4/82 (5) | 0.18 |
| Systemic lupus erythematosus | 3/157 (2) | 0 | 0.55 |
| Scleroderma | 2/157 (1) | 1/82 (1) | > 0.99 |
| Hypothyroidism | 14/157 (9) | 9/82 (11) | 0.65 |
| Sarcoidosis | 1/157 (0.6) | 4/82 (5) | 0.05 |
| Malignancy^c^ | 13/157 (8)^d^ | 8/82 (10)^e^ | 0.81 |
| Abbreviations: IBM = inclusion body myositis; ENMC = European Neuromuscular Center. Values are no. (%) unless otherwise indicated.  ^a^ Bonferroni corrected significance thresholds for clinical features were 0.003. ^b^ Five patients had two autoimmune diseases (one patient had Sjögren syndrome and systemic lupus erythematosus, one patient had rheumatoid arthritis and systemic lupus erythematosus, one patient had rheumatoid arthritis and hypothyroidism, and two patients had Sjögren syndrome and hypothyroidism). ^c^ Not all the patients were tested for CT, PET, or tumor markers as cancer screening. ^d^ Breast cancer (n = 2), prostate cancer, thyroid cancer, parathyroid adenoma, hepatocellular cancer, Paget's disease, non-Hodgkin's lymphoma, lymphoplasmacytic lymphoma, Kaposi's sarcoma, basal cell carcinoma of the forehead, small cell carcinoma of right main bronchus, and squamous cell carcinoma of unknown primary. ^e^T-cell large granular lymphocyte leukemia (n = 2), prostate cancer (n = 2), meningioma, Hodgkin's lymphoma, basal cell carcinoma in legs, and basal cell carcinoma of skin. | | | |

**Supplementary Figure 1.**


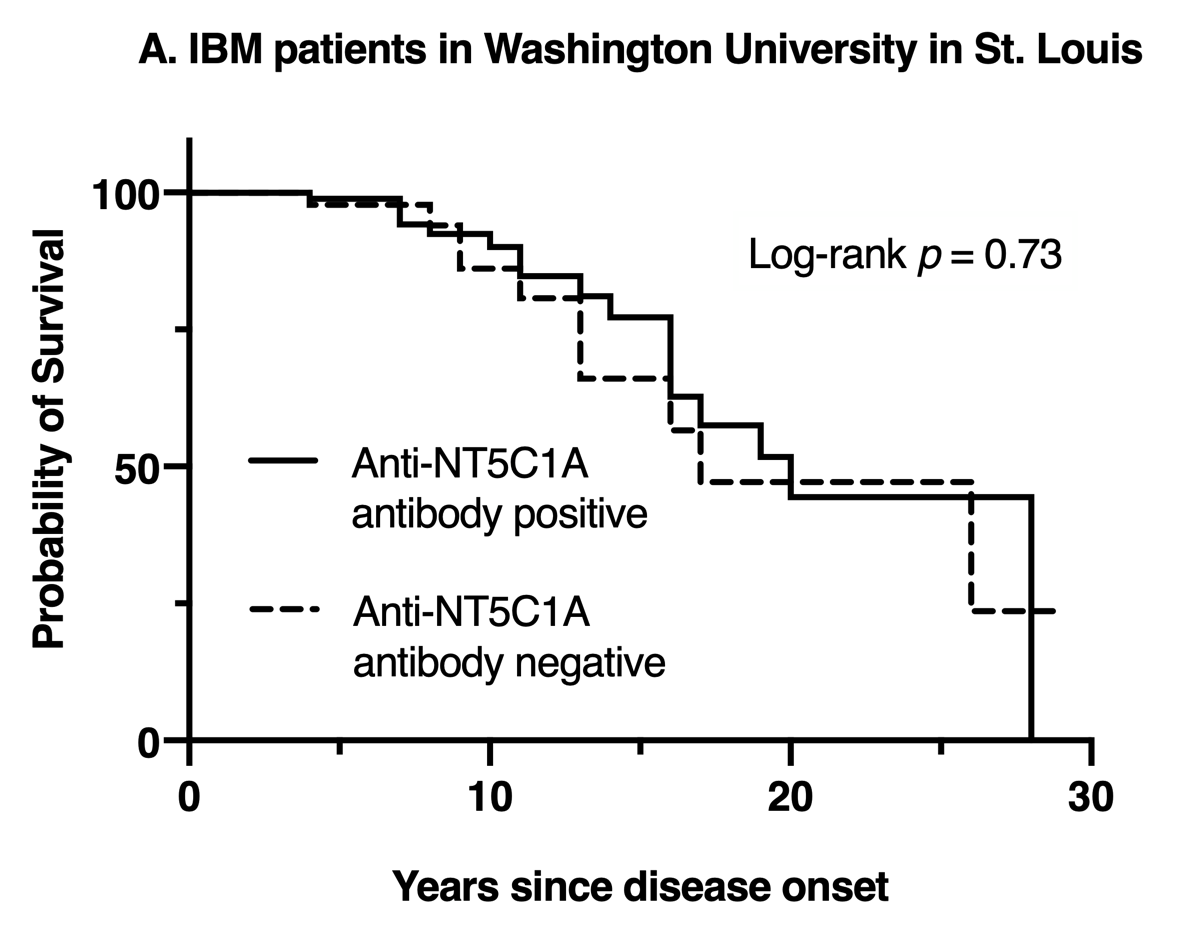


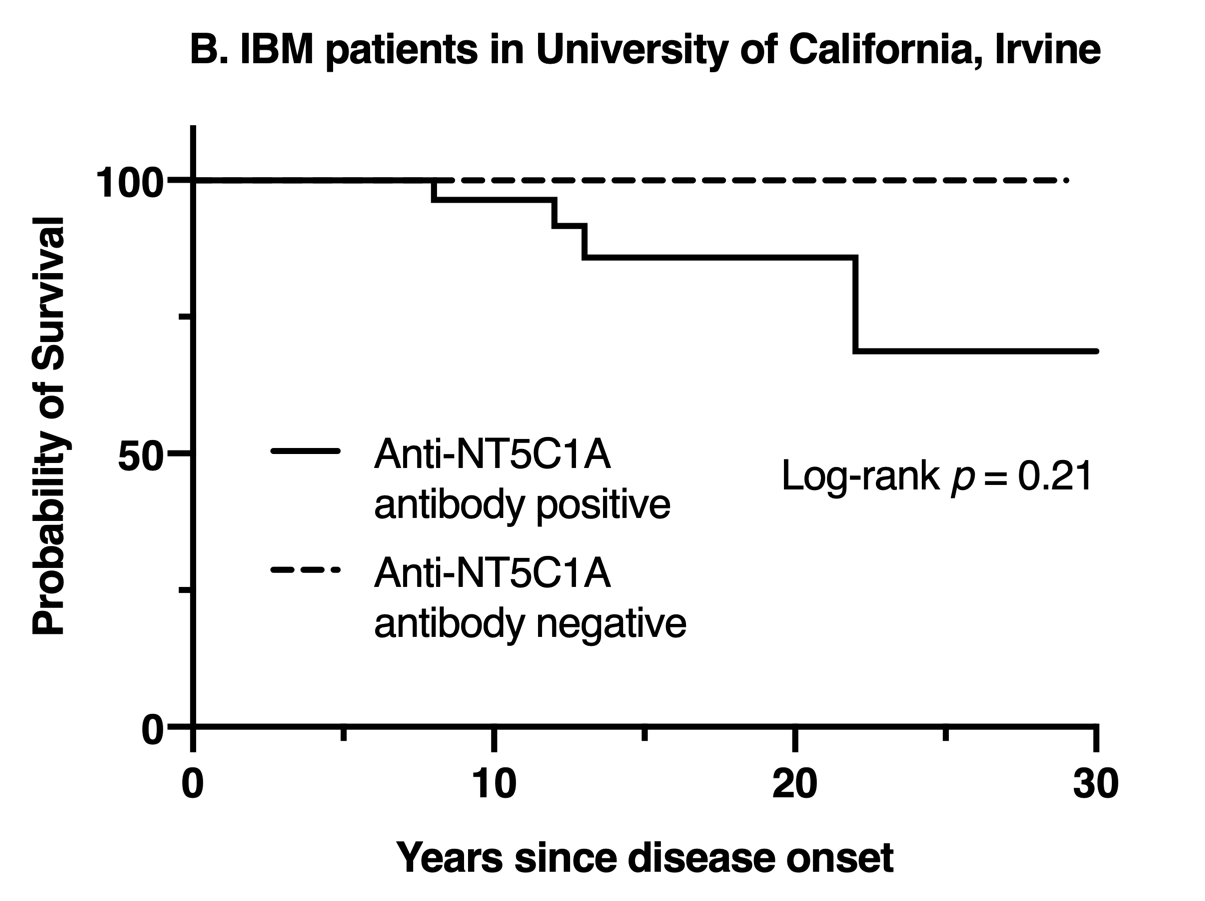

Supplement: Supplementary file 1 — Figure S1. Kaplan–Meier survival curves depending on the anti‐NT5C1A antibody status in patients with inclusion body myositis. A: Kaplan–Meier survival curves of 154 patients in the Washington University in St. Louis. B: Kaplan–Meier survival curves of 58 patients in the University of California, Irvine. Table S1. Clinicopathologic features of patients with IBM compared with non‐IBM patients with idiopathic inflammatory myopathies Table S2. Clinicopathologic features of patients with dermatomyositis, antisynthetase syndrome, and immune‐mediated necrotizing myopathy according to anti‐NT5C1A antibody status Table S3. Diagnoses with ENMC criteria and complications of patients with IBM according to anti‐NT5C1A antibody status [file ACN3-8-571-s001.docx]
